# Idiosyncratic Reaction Causing a Rare Side Effect: Isotretenoin-induced Pancreatitis

**DOI:** 10.7759/cureus.6102

**Published:** 2019-11-08

**Authors:** Muhammad Umair Atiq, Ahmad Raza, Ammar Ashfaq

**Affiliations:** 1 Internal Medicine, Abington Hospital-Jefferson Health, Abington, USA

**Keywords:** pancreatitis, isotretinoin, idiosyncratic reaction

## Abstract

Isotretinoin is a frequently prescribed medication for severe nodulocystic acne. It is also used in higher doses and other forms to treat some carcinomas. Pancreatitis remains a well-known but rare side effect of this medication. Two proposed mechanisms for pancreatitis are hypertriglyceridemia induced and idiosyncratic reaction. Here, we present a case of a young man who presented for the evaluation of abdominal pain. His blood work showed elevated lipase levels but computed tomography (CT) of the abdomen did not show any pancreatic inflammation.

## Introduction

Acute pancreatitis has an incidence of about 4.9 to 35 per 100,000 population in the US [1]. In one study, it was determined as the number one diagnosis on discharge for gastrointestinal-related admissions and was among the top 25 discharge diagnosis [2]. The clinical spectrum of acute pancreatitis can vary from mild to severe disease. Its overall mortality can be 4%, but in severe pancreatitis, complicated by pancreatic necrosis, mortality of 6%-17% has been quoted [3]. The treatment of the underlying etiology, if evident, is the cornerstone of management, as this approach also prevents recurrent episodes.

The most common causes of acute pancreatitis are gallstones (40%-70% of cases) [4], alcohol (25%-35% of cases) [5], hypertriglyceridemia (1%-14% of cases) [6], post endoscopic retrograde cholangiopancreatography (ERCP) (3%-35% of cases, depending on the type of ERCP being performed), and idiopathic (15%-25% of cases) [7]. Other less common and rare causes include certain infections (viral, fungal, bacterial, and parasitic), biliary obstruction, hypercalcemia, vascular causes, anatomic abnormalities, and medications. Medications as a cause of acute pancreatitis are implicated in less than 5% of cases [8]. Drug-induced pancreatitis (DIP) usually has a good prognosis; hence, it is vital to recognize the offending agent and stop it. DIP results from a number of mechanisms. Understanding the pathophysiology can sometimes help with identifying the population at risk. Knowledge of DIP comes mostly from case reports. The quality of evidence is not high because all the potential contributing factors are not always explored; hence, it becomes difficult to establish causality [9]. To establish causation, patients can be rechallenged with the same medication. This method might not always be ethical except when the drug is life-saving, and no other alternative is available.

DIP is classified into four categories. Category I and II drugs have more evidence in the literature as causative agents, whereas category III and IV have some supportive evidence that is not very consistent. Isotretinoin is listed under category III and has been reported as a rare cause of DIP. It is used for severe nodulocystic acne. It has also been used in some trials for cutaneous T-cell lymphoma and the prevention of squamous cell cancer of the skin in a high-risk population. It has an extensive side-effect profile with more tolerable and commonly reported cases of cheilitis to rarely reported cases of acute pancreatitis. Pancreatitis secondary to isotretinoin is proposed to be due to isotretinoin-induced hypertriglyceridemia or an idiosyncratic reaction [9]. The earlier mechanism forms the basis of monitoring triglyceride levels in patients on therapy [10]. Triglyceride levels above 500 mg/dl confer some risk of precipitating acute pancreatitis although the higher risk is above 2000 mg/dl when fasting. We present a young male who was using isotretinoin for acne and presented to the emergency department with severe abdominal pain. He met the criteria for acute pancreatitis and was treated accordingly.

## Case presentation

A 29-year-old male patient with a past medical history of Behcet's disease and acne presented to the emergency department (ER) for the evaluation of a sudden-onset, generalized abdominal pain. He had pressure-like, very severe abdominal pain radiating to the mid-back. His surgical history included repair of inguinal hernia and tonsillectomy. Family history was pertinent for diabetes and thrombocytosis in the father. The patient denied smoking, drinking, or illicit drug use. He also denied any recent sexual contacts. Vitals in the ER showed blood pressure of 115/64 mmHg, a pulse of 73 beats/min regular, a respiratory rate of 18/min, a temperature of 97.8 F measured orally, and he was saturating at 98% while breathing ambient air. Examination revealed a slim male. The cardiopulmonary exam was unremarkable. Abdominal examination revealed a soft, non-distended abdomen that had generalized tenderness, more marked in the epigastric region and right upper quadrant. Bowel sounds were present. The exam was otherwise unremarkable.

Admission labs showed blood urea nitrogen (BUN): 12, Cr: 0.72, Na: 140 mEQ/L, K: 3.8 mEQ/L, chloride: 106 mEQ/L, bicarbonate: 22 mEQ/L, aspartate aminotransferase (AST): 16, alanine aminotransferase (ALT), alkaline phosphatase (ALP): 71, total bilirubin: 0.4, calcium: 9.5 mg/dl, international normalized ratio (INR): 1.2, partial thromboplastin time (PTT): 36, white blood cell (WBC): 11.9 K/UL, hemoglobin: 14.6 gm/dl, platelets: 156 K/UL, lipase: 10350, and serum alcohol level: <10 mg/dl.

**Figure 1:**
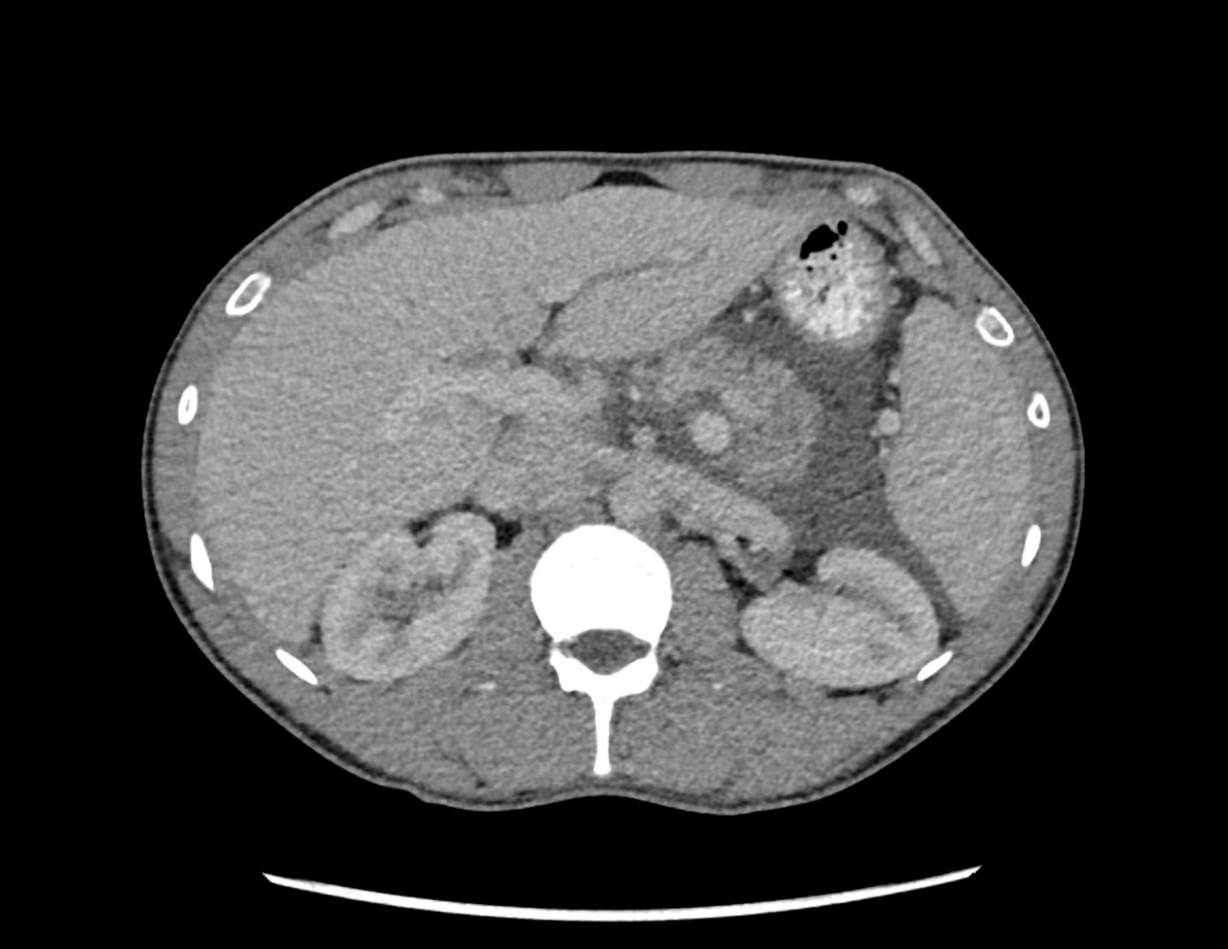
Cross-section of CT scan abdomen showing pancreas with no inflammation CT: computed tomography

Ultrasound abdomen showed no gallstones, extra or intrahepatic biliary dilatation, or any biliary obstruction. There was some perihepatic ascites. Computed tomography (CT) abdomen and pelvis with intravenous (IV) contrast showed mild intraabdominal ascites without evidence of pancreatic inflammation (Figure 1).

**Figure 2:**
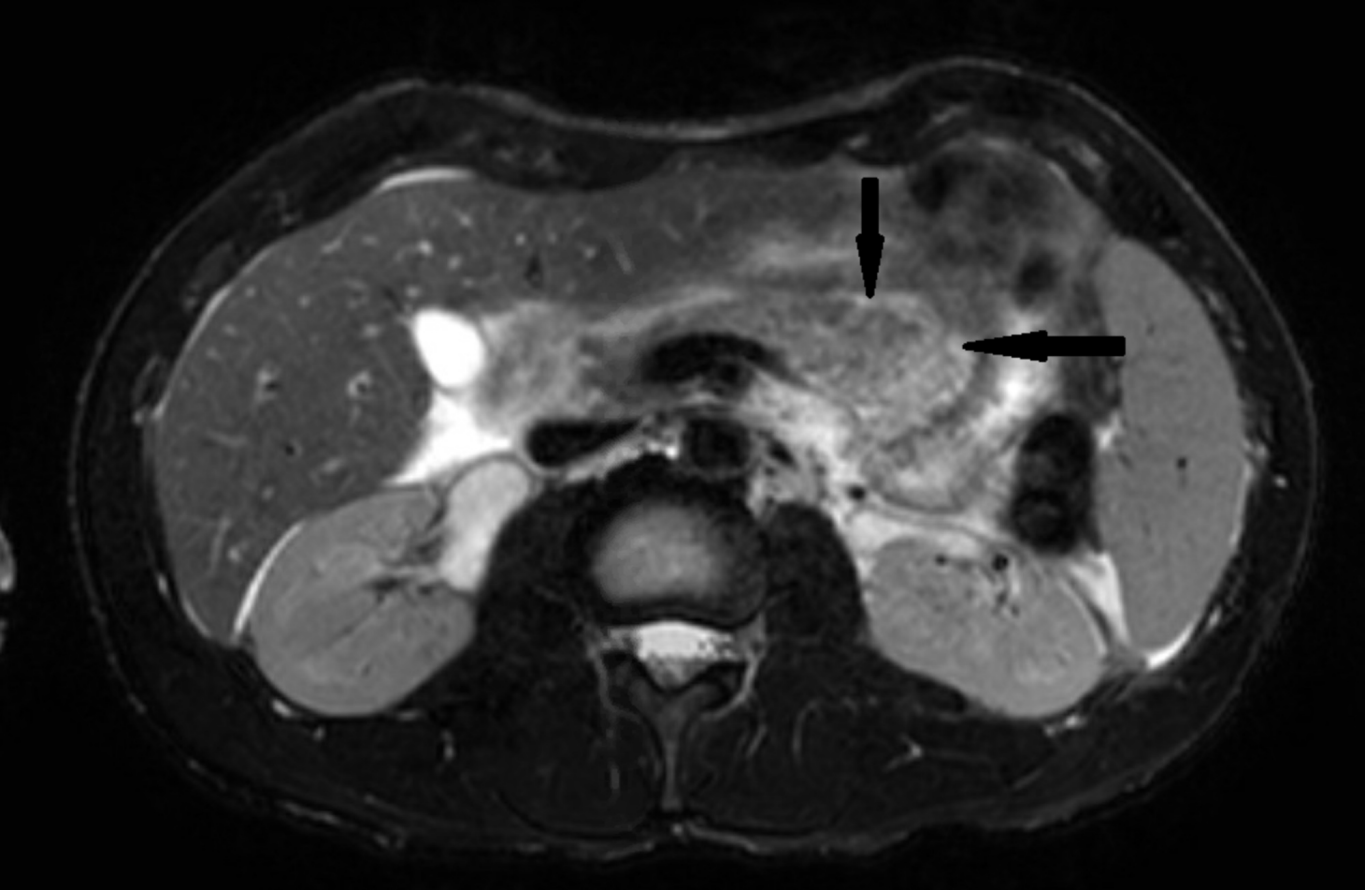
Cross-section of pancreas on MRCP abdomen. Inflammation shown by arrows. MRCP: magnetic resonance cholangiopancreatography

**Figure 3:**
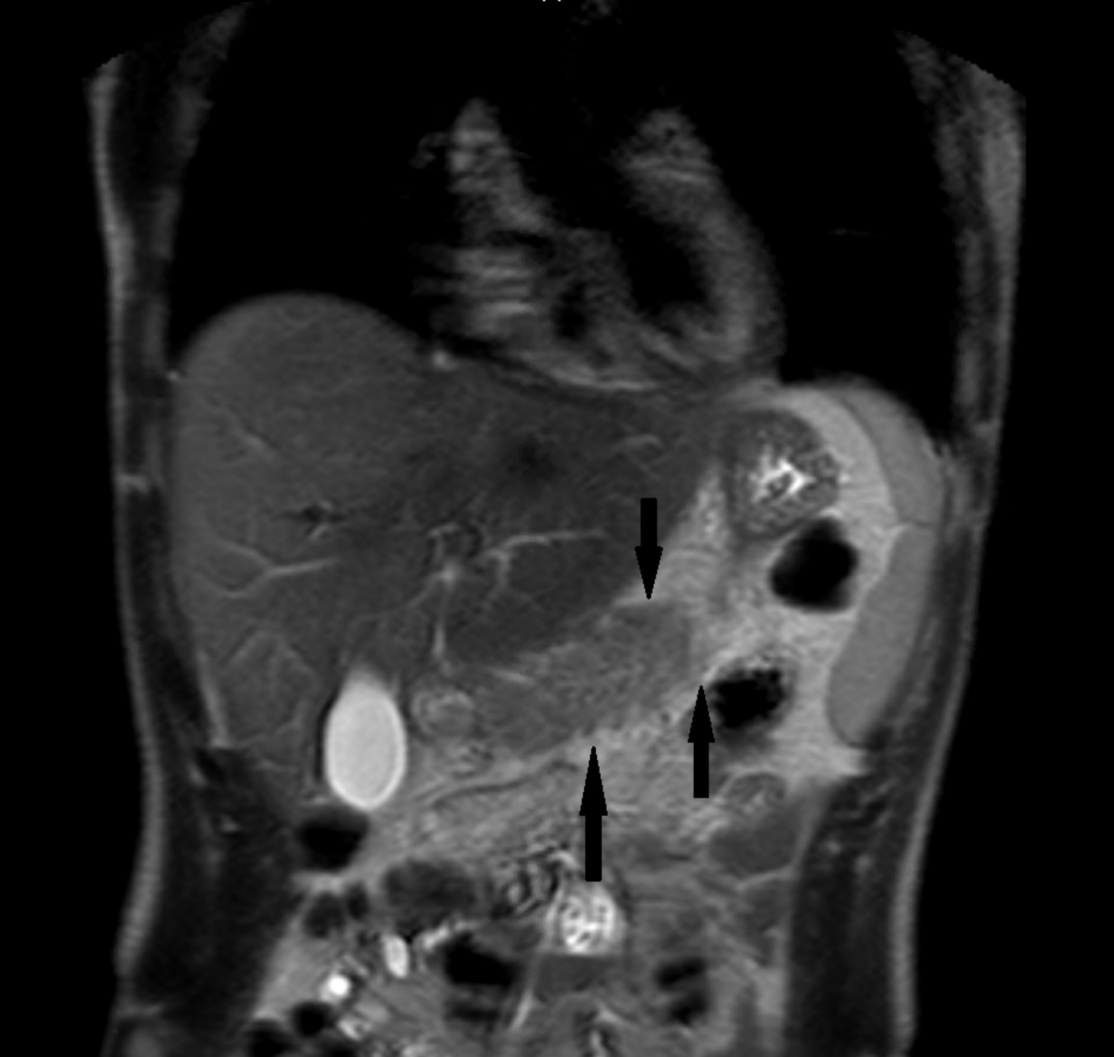
Coronal-section of the pancreas on MRCP abdomen. Inflammation shown by arrows. MRCP: magnetic resonance cholangiopancreatography

The patient was admitted and treated with IV fluids, pain medications, and was made NPO initially. Additional work-up to find the cause of pancreatitis showed cholesterol: 121 mg/dl, triglyceride: 61 mg/dl, high density lipoprotein (HDL): 26 mg/dl, very low density lipoprotein (VLDL): 12 mg/dl, low density lipoprotein (LDL): 83 mg/dl, and immunoglobulin G4 (IgG4): <0.5. The antinuclear antibody (ANA) screen was negative. Magnetic resonance cholangiopancreatography (MRCP) of the abdomen showed inflammation of the pancreas (Figure 2 and Figure 3) and mild ascites.

On admission, the patient was taking fish oil capsules, fluticasone nasal spray, isotretinoin 30 mg oral capsule, one capsule two times a day, apremilast 30 mg one tablet two times a day, and vitamin D3 1000 IU once a day.

Isotretinoin was discontinued on admission. The patient was started on a diet in almost 24 hours, and he tolerated it well without any exacerbation of symptoms. He was kept under observation and no complications were observed. Eventually, he was discharged on Day 5 of his hospital stay to follow-up as an outpatient. Symptoms had completely resolved by the time of discharge. He was advised not to restart isotretinoin upon discharge.

## Discussion

Acute pancreatitis has significant potential for morbidity and mortality, which is why identifying the underlying cause is crucial. Isotretinoin causing DIP through hypertriglyceridemia is rare. The natural history of triglyceride elevation while on isotretinoin is predictable to some extent and can be used to help guide the monitoring of lipid levels. The pattern of elevation, unfortunately, does not predict the timing of DIP except that it does not happen within six to seven weeks of initiation of isotretinoin [10]. In a retrospective trial of patients taking isotretinoin, the incidence of lipid disorders, including hypertriglyceridemia, was up to 3.11%. The trial did not report any case of acute pancreatitis [11]. Four cases have been reported so far in literature linked to hypertriglyceridemia. One of the cases involved the treatment of glioblastoma with the use of high-dose isotretinoin [12]. The rest are case reports with cases who had elevated baseline triglyceride levels and, subsequently, had a very high rise in triglyceride levels. One of the patients, although had a previous history of very high triglyceride levels (4040 mg/dl), did not develop acute pancreatitis previously [13]. Two of the case reports had patients on conjugated estrogen, tetracycline, and prednisone, all of which can potentially cause acute pancreatitis [9,14-15].

Idiosyncratic, in contrast to triglyceride induced, has no predictable pattern, cannot be monitored, and is also a rare entity in the literature. Twenty-one cases have been reported as causing DIP without raised triglyceride levels. Sixteen cases come from a French pharmacovigilance report, among which only seven patients had triglyceride levels [16]. Only one patient had a slightly elevated triglyceride level. Two patients were also on oral contraceptive pills (OCPs) and one of them had alcohol as a confounding factor. Among the rest of the five cases, four cases are from cohort studies for the treatment of various diseases, and the remaining is a case report of a patient treated for acne. One of the patients was also on estradiol [17]. At least one of the patients was on interferon-alpha [18], which has also been implicated as category III for DIP [9]. Two other reported patients had gallstones. One of them had a second episode while on isotretinoin and had biliary slush detected during the second episode. This patient was reported with the 16 cases in the French report originally [19-20]. Including the above-mentioned case from the French pharmacovigilance report and five other cases reported elsewhere, five out of six patients had no baseline triglyceride levels. Only three out of six patients had peak triglyceride levels reported, which makes it hard to establish if all the cases resulted from an idiosyncratic reaction. The total time that patients were on isotretinoin ranged from 10 days to one year, including cases for both triglyceride-induced and idiosyncratic pancreatitis.

In our patient, triglyceride levels were within normal limits without the use of any lipid-lowering agent; hence, we are inclined to believe that this was most likely an idiosyncratic reaction. Idiosyncratic reactions, as mentioned before, are reported to occur at a variable time during treatment. Severe pain while being on isotretinoin therapy warrants further exploration, and patients should be aware of this complication. This diagnosis, however, should be made carefully and after the exclusion of common causes.

## Conclusions

This case reminds us of the importance of a detailed medication list review while evaluating a patient with pancreatitis. While it can be relatively easy to avoid the culprit medication to prevent further attacks, it can still be challenging to find a suitable and equally effective alternative, especially for patients in absolute need of those medications.
